# Exercise-induced hypothalamic neuroplasticity: Implications for energy and glucose metabolism

**DOI:** 10.1016/j.molmet.2023.101745

**Published:** 2023-05-31

**Authors:** Eunsang Hwang, Bryan Portillo, Kyle Grose, Teppei Fujikawa, Kevin W. Williams

**Affiliations:** Center for Hypothalamic Research, the University of Texas Southwestern Medical Center at Dallas, Dallas, TX, USA

**Keywords:** hypothalamus, exercise, physical activity, neuroplasticity, exerkine, melanocortin

## Abstract

**Background:**

Neuroplasticity refers to the brain's ability to undergo functional and structural changes in response to diverse challenges. Converging evidence supports the notion that exercise serves as a metabolic challenge, triggering the release of multiple factors both in the periphery and within the brain. These factors actively contribute to plasticity in the brain, and in turn, regulate energy and glucose metabolism.

**Scope of Review:**

The primary focus of this review is to explore the impact of exercise-induced plasticity in the brain on metabolic homeostasis, with an emphasis on the role of the hypothalamus in this process. Additionally, the review provides an overview of various factors induced by exercise that contribute to energy balance and glucose metabolism. Notably, these factors exert their effects, at least in part, through actions within the hypothalamus and more broadly in the central nervous system.

**Major Conclusions:**

Exercise elicits both transient and sustained changes in metabolism, accompanied by changes in neural activity within specific brain regions. Importantly, the contribution of exercise-induced plasticity and the underlying mechanisms by which neuroplasticity influences the effects of exercise are not well understood. Recent work has begun to overcome this gap in knowledge by examining the complex interactions of exercise-induced factors which alter neural circuit properties to influence metabolism.

## Abbreviations

aBNST: Bed nucleus of the stria terminalis
AD: Alzheimer's disease
ARC: Arcuate nucleus
βAdRs: β-adrenergic receptors
BAT: Brown adipose tissue
BDNF: Brain-derived neurotrophic factor
CEA: Central nucleus of the amygdala
CNDP2: Cytosolic nonspecific di-peptidase 2
CNS: Central nervous system
CRF: Corticotrophin-releasing factor
DBHB: d-β-hydroxybutyrate
DIO: Diet induced obese
DMH: Dorsal medial hypothalamus
DMV: Dorsal motor nucleus of the vagus
GHSR: Growth hormone secretagogue receptor
GLP-1R: Glucagon-like Peptide-1 receptor
HDAC: Histone deacetylase
HIIE: High intensity interval exercise
ICV: Intracerebroventricular
IL-6: Interleukin 6
Lac-Phe: N-lactoyl-phenylalanine
LEAP2: Liver-expressed antimicrobial peptide 2
LepR: Leptin receptor
LH: Lateral hypothalamus
MC4R: Melanocortin-4 receptor
MnPO: Median preoptic nucleus
NAFLD: Non-alcoholic fatty liver disease
NPY/AgRP: Neuropeptide Y/agouti-related peptide
NTS: Nucleus of the Solitary Tract
PACAP: Pituitary adenylate-cyclase-activating polypeptide
PAG: Periaqueductal grey
PNOC: Prepronociceptin
POMC: Proopiomelanocortin
POA: Preoptic areas
PPG: Preproglucagon
PVH: Paraventricular hypothalamus
PVT: Paraventricular thalamic nucleus
SF1: Steroidogenic factor 1
SHy: Septohypothalamic nucleus
Sim1: Single-minded 1
SNS: Sympathetic nervous system
SON: Supraoptic nucleus
SST: Somatostatin
T2D: Type 2 diabetes
TH: Tyrosine hydroxylase
TRH: Thyrotropin releasing hormone
TRPV1: Capsaicin-sensitive transient receptor potential vanilloid 1
TTR: Transthyretin
VMH: Ventral medial hypothalamus
WAT: White adipose tissue

## 1 Introduction

The increasing prevalence of metabolic diseases is an economic burden on health systems. Excessive consumption of calorically dense food and a sedentary lifestyle increases the incidence of obesity. Obesity, in turn, is associated with a variety of diseases, including type 2 diabetes, non-alcoholic fatty liver disease, and coronary heart disease, which can result in premature death. Lifestyle modifications, such as increased physical activity and/or exercise, are frequently recommended for patients with obesity, diabetes, and vascular disease in order to improve and/or prevent various medical complications.

**Figure 1:**
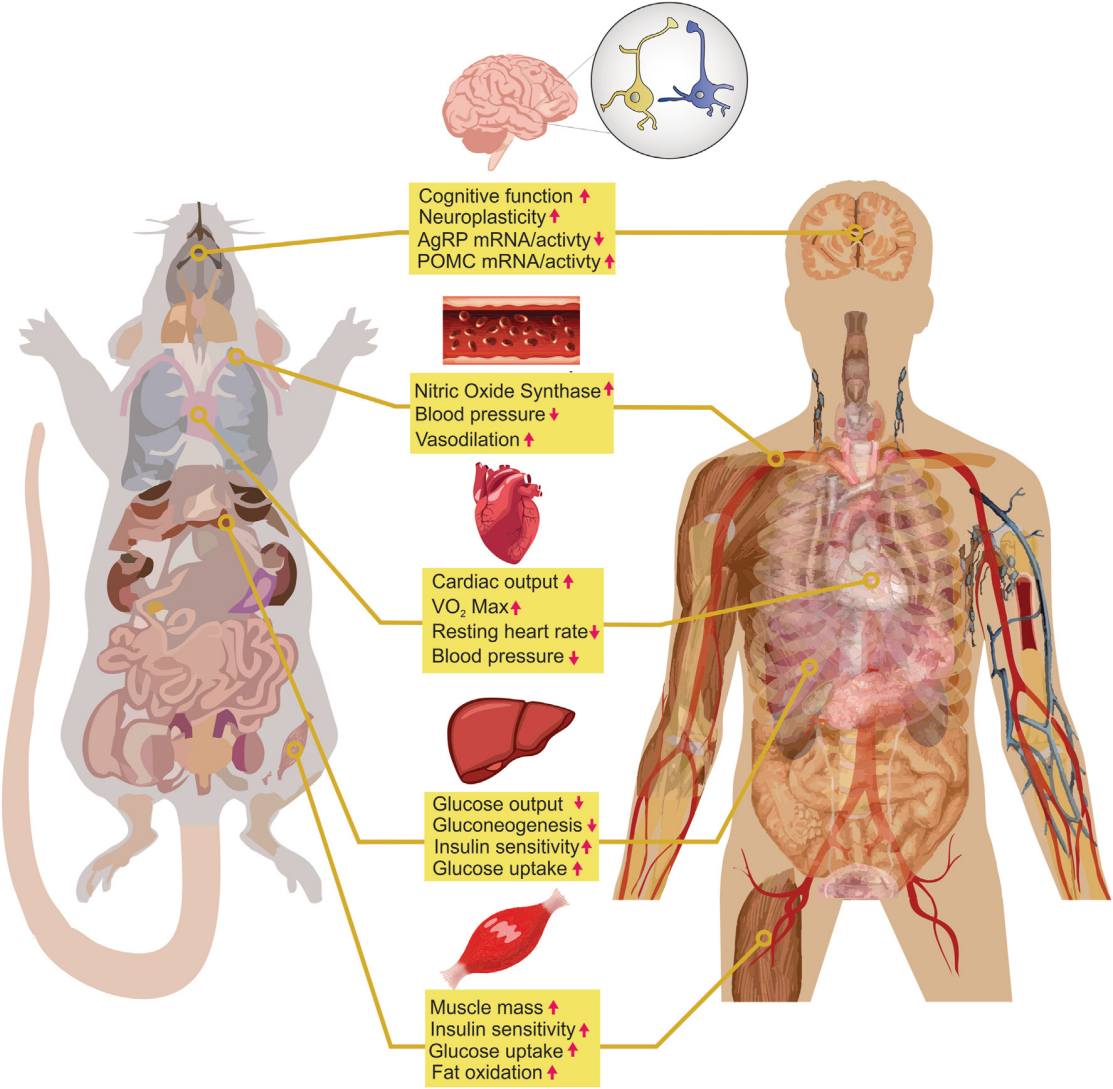
**Exercise-related health benefits.** Shown here are improvements in response to exercise in multiple organs. These exercise-induced changes have been linked to reducing the presence or severity of disease.

Exercise (single bout and/or chronic training) is associated with a wide range of benefits, including improved cardiovascular function [1], enhanced learning and memory [[2], [3], [4]], and improved metabolism [5,6] (Figure 1). In particular, exercise training promotes vasodilation, nitric oxide synthase, and functional adaptation of the heart [7,8], leading to improved cardiovascular health. In rodents, exercise has been shown to enhance hippocampal-associated learning and memory tests [2,3]. Similarly, in humans, exercise has been shown to delay age-related cognitive decline [9,10].

Metabolic improvements in response to exercise include increased insulin sensitivity, which leads to enhanced glucose uptake in muscles and decreased glucose production in the liver [11,12]. These benefits have been observed in both lean and obese individuals and may persist for several days following a single bout of exercise [11,12]. High-intensity exercise or high work-load exercise can also acutely decrease hunger/feeding in the hours immediately post-exercise in multiple species, whereas mild to moderate exercise may stimulate appetite to compensate for the caloric demands of exercise [[13], [14], [15]]. While some of these effects are due to direct actions in the periphery, the plasticity of the central nervous system (CNS) also plays a role in metabolic improvements following exercise [16]. This review highlights emerging findings that illustrate how exercise modifies neuron circuits in the brain, and its implications on metabolism (with an emphasis on action within the hypothalamus).

## 2 Exercise and the hypothalamus

Over the past century, extensive experimental evidence has consistently highlighted the brain, particularly the hypothalamus, in regulating body weight and blood glucose levels [[17], [18], [19], [20], [21], [22]]. Clinical reports of hypothalamic insufficiency resulting from tumors of the pituitary have supported this understanding [17,[22], [23], [24]]. Animal models with targeted lesions in specific regions, nuclei, or cell populations in the hypothalamus have replicated the observed clinical manifestations observed in humans, providing additional evidence. Moreover, molecular genetics, in conjunction with methods to monitor and manipulate neuronal activity in a cell-specific manner, has identified multiple nuclei and cell populations within the hypothalamus that contribute to energy and glucose homeostasis [17,19,20,22,25]. Key hypothalamic nuclei include the lateral hypothalamus (LH), paraventricular hypothalamus (PVH), ventral medial hypothalamus (VMH), dorsal medial hypothalamus (DMH), and arcuate nucleus (ARC). These hypothalamic nuclei and associated cell populations are sensitive to changes in nutrient availability and circulating factors that influence food intake, body weight, and blood glucose regulation [17,19,22,[25], [26], [27], [28]]. Neuronal activity in these regions is rapidly modulated in response to food consumption with specificity to particular nutrient substrates. Moreover, the activity state of these neurons directly influences feeding behavior, blood glucose levels, and substrate utilization [17,19,[29], [30], [31], [32], [33], [34], [35]]. These findings suggest that hypothalamic nuclei, in response to various metabolic challenges, can rapidly alter neuronal activity in a sustained manner in order to influence energy and glucose metabolism.

Exercise is also a metabolic challenge that results in rapid and/or sustained changes in energy and glucose homeostasis [5,19,[36], [37], [38], [39], [40], [41]]. This has sparked considerable interest in understanding how exercise modifies the activity of metabolically relevant neurons in the brain [19,35,[42], [43], [44]]. Recent work has focused on exercise-induced plasticity of identified metabolically-relevant neurons in the hypothalamus, including the arcuate nucleus (ARC), ventral medial hypothalamus (VMH), dorsal medial hypothalamus (DMH), and paraventricular hypothalamus (PVH).

These studies aim to unravel how exercise influences energy and glucose metabolism by studying the plasticity of these metabolically-relevant neurons and their associated neural circuits in the brain. Importantly, the conservation of these hypothalamic regions, which play a crucial role in regulating various aspects of energy and glucose metabolism across species, highlights the potential significance of this knowledge in deepening our understanding of how exercise impacts human health and well-being [45,46].

This review discusses results from multiple species, including rodents and humans. Species information is provided where appropriate. However, unless specifically stated otherwise, the review predominantly relies on findings from rodent experiments.

### 2.1 The arcuate nucleus (ARC)

The hypothalamic arcuate nucleus (ARC) plays a crucial role in regulating metabolism by receiving input from peripheral hormones that signal energy storage and nutrient availability [18,21,[47], [48], [49], [50]]. Subsequently, the ARC relays this information to various brain regions, both within and outside the hypothalamus, leading to altered metabolism [18,21,25,45,48,49,51]. Among the diverse cell types within the ARC, two notable populations involved in this regulatory process are the anorexigenic Proopiomelanocortin (POMC) neurons and the orexigenic Neuropeptide Y (NPY)/agouti-related peptide (AgRP) neurons. Activation of ARC NPY/AgRP and/or a rise in central NPY or AgRP levels is sufficient to stimulate foraging behavior, promote feeding, reduce energy expenditure, and impair glucose metabolism in mice [31,46,[52], [53], [54], [55]]. Conversely, activation of ARC POMC neurons or administration of the biologically active peptide alpha-melanocyte-stimulating hormone (α-MSH) that is derived from POMC, has been found to suppress feeding, increase energy expenditure, and improve glucose metabolism in rodents [45,56,57]. Electrophysiology and optical imaging further demonstrated that ARC NPY/AgRP and POMC neurons rapidly reorganize their activity state in response to nutrients, hormones, and neurotransmitters [19,44,[58], [59], [60], [61], [62]]. This reorganization can be either transient or sustained, giving rise to a plasticity of this cell population to influence metabolism. These recent data highlight the ability of ARC POMC and NPY/AgRP neurons to acutely and chronically alter their activity state in order to modify energy and glucose homeostasis in response to variable energy needs.

**Figure 2:**
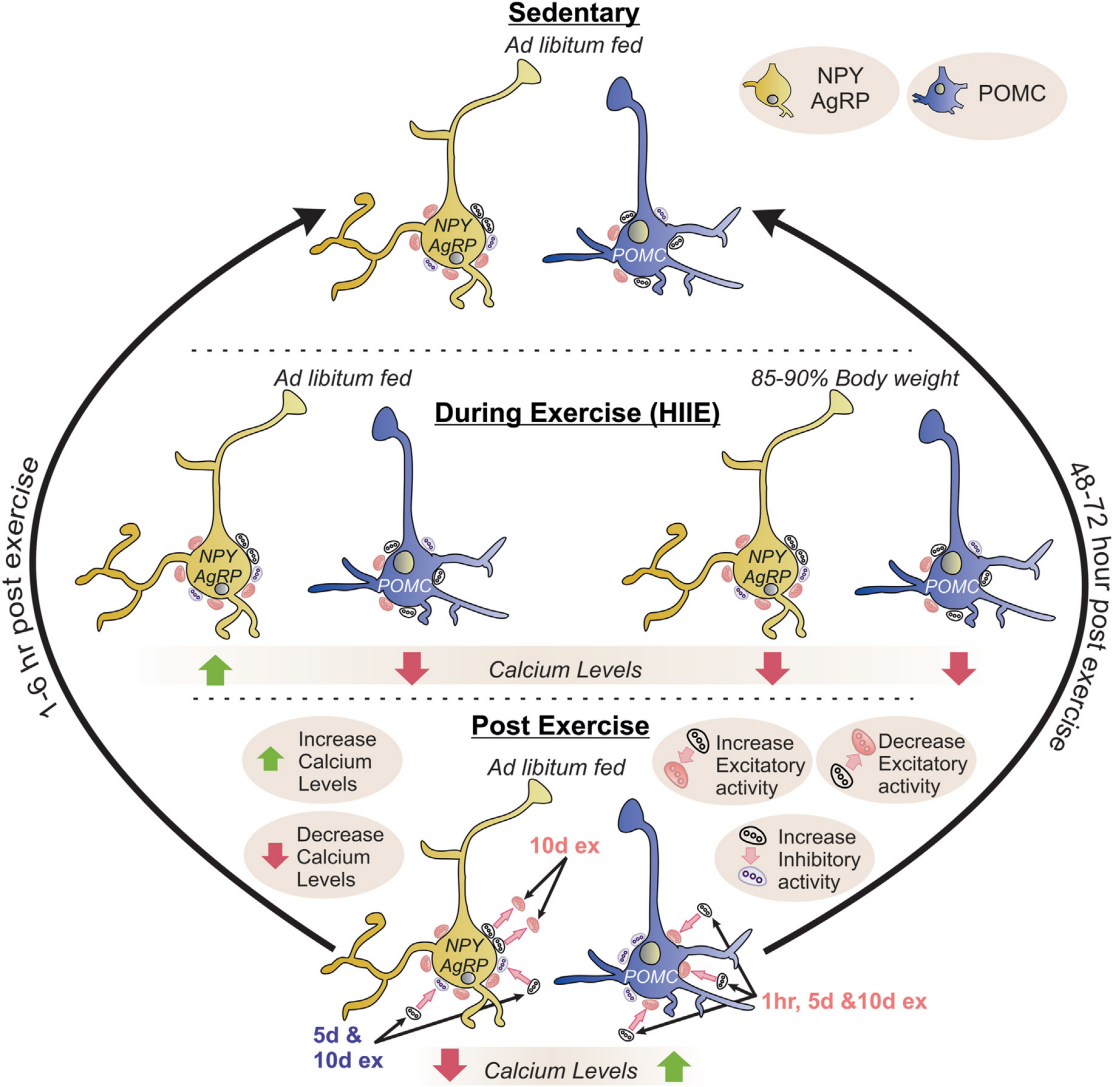
**State dependent changes of NPY/AgRP and POMC neurons during and after exercise – involvement in neuroplasticity.** The effects of exercise under different nutrient states on arcuate NPY/AgRP and POMC neuron activity is summarized from top to bottom (top: sedentary state, middle: during exercise, bottom: post exercise). The yellow cell represents an NPY/AgRP neuron, and the blue cell represents a POMC neuron. Inhibitory and excitatory synaptic inputs are represented by gray and red elliptical shapes on the surface of each neuron, respectively. The green arrow represents an increase in calcium activity and the red arrow represents a decrease in calcium activity. NPY/AgRP calcium activity during exercise is dependent on the nutrient state of the mouse; italicized as either ad libitum fed or held at 85–90% body weight. The straight pink arrows are pointing to changes in synaptic activity (either increased or decreased). The straight black arrows (in post exercise) are showing the time frame for these synaptic changes to happen. The black curved arrows from the post exercise to sedentary state indicate the duration that these synaptic changes persist following a bout of exercise before returning to the sedentary state (1 h–72 h later).

The effects of exercise on the activity state and plasticity of ARC POMC and NPY/AgRP neurons have traditionally been poorly understood. However, recent work has demonstrated that ARC POMC neurons are activated following high-intensity interval exercise (HIIE) in mice, while ARC NPY/AgRP neurons are inhibited [43] (Figure 2). A similar inhibition of ARC NPY/AgRP neurons was observed after voluntary exercise in mice [63]. Interestingly, the activity states of ARC POMC and NPY/AgRP neurons are temporally variable following forced HIIE exercise, with the inhibition of NPY/AgRP neurons lasting for only a few hours, while the exercise-induced activation of POMC neurons is sustained for at least 2 days after a single bout of exercise (Figure 2).

During exercise, mice on an unrestricted chow diet exhibited heightened activation of ARC AgRP neurons and decreased ARC POMC neuron activity as determined by monitoring in-vivo calcium levels via photometry recordings [19]. The level of cellular activity of ARC AgRP and POMC neurons in awake-behaving mice is dependent upon the intensity of exercise, as the level to which ARC AgRP neurons were activated and ARC POMC neurons were inhibited was directly proportional to the speed of running. Interestingly, exercise-induced inhibition of both AgRP and POMC neurons occurs when mice are food deprived (held at 85–90% of their initial body weight) [19], indicating the important role of nutrient availability in the exercise-induced activity state of these neurons (Figure 2).

Taken together, the activity of ARC POMC and NPY/AgRP neurons during exercise is influenced by nutrient availability and exercise intensity. In animals with unrestricted access to food, the onset of exercise leads to increased activity of AgRP neurons and inhibition of POMC neurons [19,63]. This potentially contributes to increased energy intake, improved glucose metabolism, and a preference for carbohydrate utilization [19]. This activity profile may also align with the metabolic demands of exercise in humans, which often involve increased glucose production and a preference for carbohydrate utilization particularly during high-intensity exercise [64,65]. During food deprivation, exercise leads to inhibition of both AgRP and POMC neurons, and an activity profile indicative of an acute decrease in a drive for energy intake. These acute changes can still result in increased glucose production and decreased carbohydrate utilization or preference compared to fat utilization [19,[63], [64], [65]]. As increased ARC POMC neuronal signaling improves insulin sensitivity and glucose metabolism, the sustained activation of ARC POMC neurons after exercise aligns with improved insulin sensitivity observed for up to 48 h after a single bout of exercise which is observed across various species [[38], [39], [40], [41]]. Furthermore, since NPY/AgRP neurons play a role in regulating acute feeding behavior [19,31,52,55], the transient suppression of NPY/AgRP neuronal activity after exercise may correlate with the acute hypophagia (reduced food intake) observed following high-intensity exercise. This is supported by the suppression of food intake in mice immediately after a single bout of HIIE [43].

It is important to note that the ARC is not exclusively comprised of NPY/AgRP and POMC neurons. There are multiple other cell populations within the ARC that have also been implicated in energy and glucose metabolism. One such population is the GABAergic non-AgRP, non-POMC neurons, which play a role in mediating some of the effects of leptin on energy balance [[66], [67], [68]]. Whether this GABAergic neuron population overlaps with tyrosine hydroxylase (TH), prepronociceptin (PNOC), or somatostatin (SST) neurons within the ARC remains to be determined. However, these additional neuron populations have also been implicated in regulating feeding behavior and body weight control [69]. Understanding the effects of exercise on these diverse cell populations within the ARC will be an important next step in fully elucidating the effects of exercise within this hypothalamic region.

### 2.2 Ventromedial nucleus of the hypothalamus (VMH)

Increasing evidence suggests that VMH steroidogenic factor 1 (*SF1*) neurons sense hormonal cues to regulate basal metabolism, primarily in response to metabolic challenges that occur with high-fat diet feeding [70]. Stimulation of the VMH or SF1 neurons increases sympathetic nervous system (SNS) outflow to mediate glucose uptake in skeletal muscle and brown adipose tissue (BAT), as well as promoting lipolysis in white adipose tissue (WAT) in rodents [71,72]. Moreover, acute chemogenetic activation of SF1 neurons enhances insulin sensitivity in the skeletal muscle of sedentary mice [73].

Exercise is a robust metabolic challenge that is also influenced by the VMH [36]. In particular, antagonism of β-adrenergic receptors (βAdRs) in the VMH of rats delays the exercise-induced increase in circulating fatty acids [74]. Moreover, lidocaine or βAdR antagonists administered directly into the VMH attenuates fatty acid oxidation during exercise [75]. VMH-specific deletion of SF-1 also blunts reductions in fat mass, reduces improvements in glycemia, and mitigates increases in energy expenditure that are associated with exercise training [76]. In addition to the beneficial effects of exercise, VMH neurons have also been implicated in the pivotal flip between sedentary and active lifestyles in female mice [77]. In particular, estrogen, via estrogen receptor alpha (ERα) in the ventrolateral VMH (vlVMH), increases melanocortin-4 receptor (MC4R) expression. Activation of these vlVMH^ERα/MC4R^ neurons promotes physical activity in mice. While pharmacogenetic activation of VMH *SF1* neurons has been shown to suppress feeding and activation of VMH *SF1* neurons or vlVMH^ERα/MC4R^ improves insulin sensitivity [29,73], the activity/plasticity of *SF1* neurons during or after exercise remains undefined. Of note, suppression of neuronal activity in the VMH apparently does not affect metabolism under sedentary conditions [75]. Glutamatergic VMH neurons are synaptically coupled to arcuate POMC and NPY/AgRP neurons [78]. As these glutamatergic VMH neurons are upstream of target neurons in the arcuate, the exercise-induced effects previously described in arcuate POMC and NPY/AgRP neurons may require, at least in part, activity-induced changes of VMH neurons [43]. These findings emphasize the need to investigate VMH cell populations to better understand how exercise influences brain plasticity and long-term metabolic changes.

### 2.3 Dorsomedial hypothalamic nucleus (DMH)

Classic lesion studies in rodents have demonstrated that the dorsomedial hypothalamic nucleus (DMH) contributes to feeding behavior, energy expenditure, and ultimately body weight regulation [79]. Activation or disinhibition of the DMH induces nonshivering thermogenesis and increases body temperature [80,81]. These effects are intertwined with circulating metabolic signals, neurotransmitters, and ultimately autonomic outflow [[82], [83], [84]]. Additionally, leptin receptor (LepR) neurons in the DMH are synaptically and functionally linked to ARC NPY/AgRP (and to a much lesser degree ARC POMC) neurons and feeding [59,85]. In particular, sensory detection and subsequent consumption of food activates DMH LepR/GABA neurons, which in turn inhibit ARC NPY/AgRP neurons [59,85]. These findings highlight the DMH as a crucial node in the brain that senses, transmits, and adjusts metabolism in response to variable energy states or requirements.

Beyond its classic role in energy regulation, the DMH has also been linked to physical activity and exercise. Voluntary wheel running activates DMH neurons, particularly in the ventral and caudal subregions [86]. This activity may be linked to corticotrophin-releasing factor (CRF), neuropeptide Y (NPY), and transthyretin (TTR) in the DMH [86,87]. Activation or disinhibition of DMH neurons promotes overall running activity (reducing fatigue) while also reducing food intake and body weight [86,88]. Similar to the VMH, the neurons (particularly in the ventral and caudal subregions) of the DMH are synaptically coupled to ARC NPY/AgRP neurons and may form a circuit (DMH LepR/GABA → ARC NPY/AgRP) co-opted during exercise and/or exercise training to facilitate proper energy metabolism.

### 2.4 Paraventricular hypothalamus (PVH)

Similar to the ARC, VMH, and DMH, classical lesion and pharmacological studies have implicated the PVH in regulating energy balance and glucose metabolism [89,90]. PVH MC4R neurons are downstream and putative effector targets of ARC POMC and NPY/AgRP neurons involved in feeding and body weight regulation [55,90,91]. In particular, PVH MC4R neurons bi-directionally regulate feeding behavior in response to artificial inhibition and activation, driving hunger and satiety, respectively [91]. The ARC POMC and NPY/AgRP → PVH MC4R neurons form a key component of the melanocortin circuit, which plays a major role in monogenic forms of obesity and diabetes across various species, including rodents and humans [92,93]. This connection makes the melanocortin circuit a crucial factor in bridging metabolic studies in animal models with those in humans.

In addition to MC4R neurons, multiple cell populations within the PVH have been implicated in feeding, blood glucose control, and body weight regulation [32,94,95]. These populations include, but are not limited to: Single Minded 1 (Sim1), Pituitary adenylate-cyclase-activating polypeptide (PACAP), thyrotropin releasing hormone (TRH), oxytocin, and Glucagon-like Peptide-1 receptor (GLP-1R) neurons. While activation of several of these neuronal populations suppresses feeding or improves energy balance [32,94,[96], [97], [98]], activation of PVH Sim1+/TRH+/PACAP+ neurons which project to arcuate AgRP neurons results in an excitatory orexigenic circuit [99].

PVH neurons also coordinate responses of skeletal muscle, WAT, and BAT to various metabolic challenges [100]. Additionally, the PVH provides an important bidirectional control of autonomic outflow, at least in part, via a reciprocal connection with the Nucleus of the Solitary Tract (NTS). The NTS receives viscerosensory information from the thoracic and abdominal viscera [101,102]. This sensory information includes gastrointestinal sensation as well as cardiovascular information from arterial mechanoreceptors (also known as baroreceptors). Thus the NTS-PVH-NTS connection acts as a real-time feedback loop for viscerosensory information that, in addition to controlling energy balance, can facilitate rapid cardiovascular adjustments [103].

Using cFos as a measure of cellular activity; acute, moderate intensity exercise activates PVH Sim1 neurons [104]. Additionally, exercise training induces structural and functional neuroplasticity of PVH neurons that project to the dorsal brainstem, including the NTS and dorsal motor nucleus of the vagus (DMV) [105,106]. The specific role of these changes in contributing to the beneficial effects on energy balance and glucose metabolism has traditionally been less understood. However, the PVH-NTS descending peptidergic pathways may play a crucial role in regulating cardiovascular function during acute bouts of exercise [106,107]. In particular, vasopressin resets reflex control from bradycardia, ultimately facilitating exercise-induced tachycardia in both sedentary and trained individuals [106]. On the other hand, oxytocin complements this activity by promoting a slower heart rate during exercise in trained individuals [106]. Thus, balanced action of vasopressin and oxytocin in the PVH-NTS circuit is necessary to effectively coordinate the cardiovascular system and adapt to the changing physiological demands of exercise.

## 3 Potential exercise-induced triggers of neural plasticity which promote the beneficial effects of exercise

**Figure 3:**
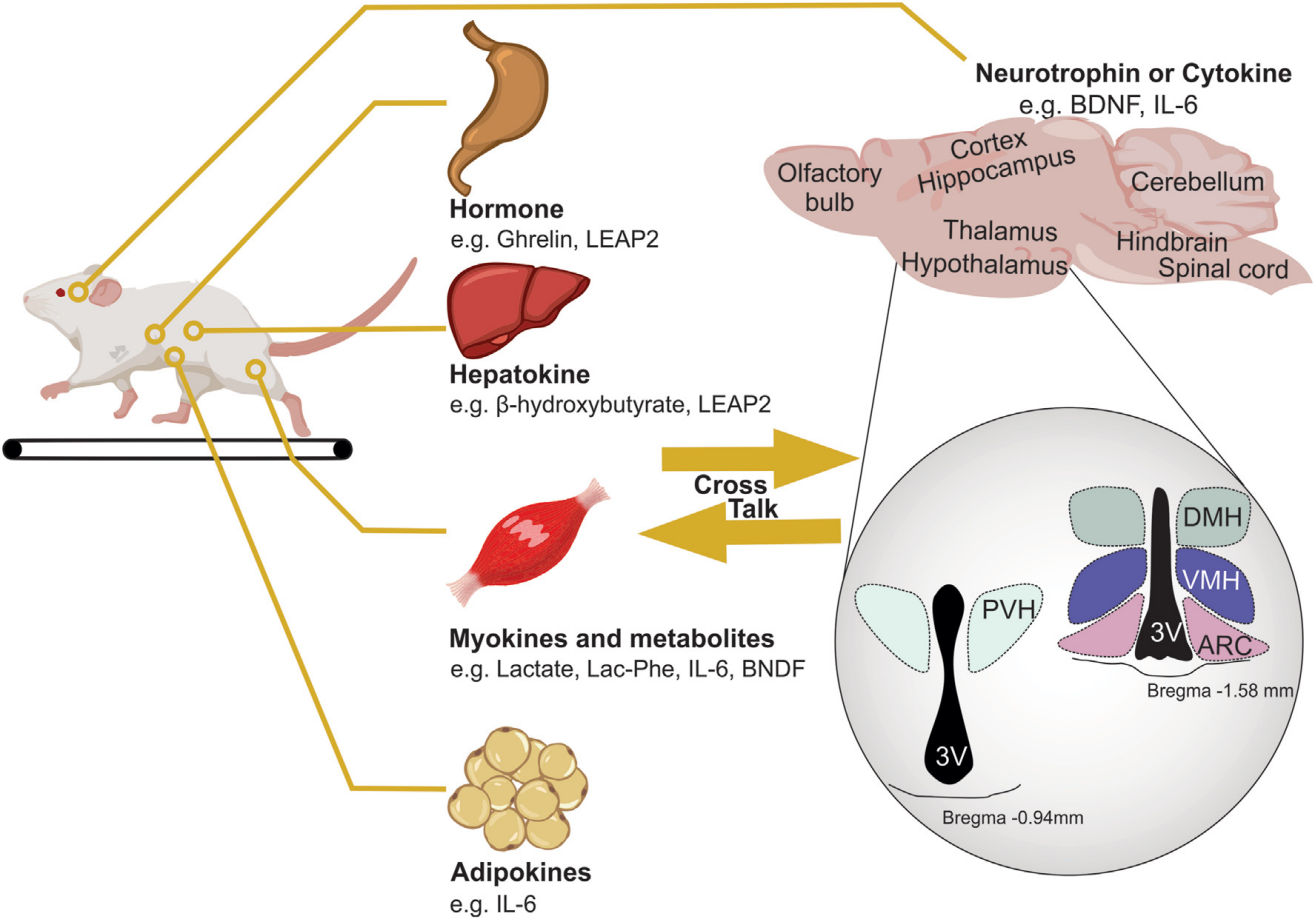
**Cross talk between the periphery and the brain leading to exercise-induced plasticity.** Shown here are organs and tissues that release hormones, hepatokines, myokines, adipokines, cytokines, metabolites and neurotrophins via exercise. These factors are linked to functional and structural changes of neural circuits within the brain (including within the hypothalamus). Lac-Phe: Lactoylphenylalanine, LEAP2: Liver-expressed antimicrobial peptide 2, BDNF: Brain-derived neurotrophic factor, IL-6: interleukin 6, PVH: Paraventricular hypothalamus, DMH: Dorsomedial hypothalamus, VMH: Ventromedial hypothalamus, ARC: Arcuate nucleus, 3V: Third ventricle.

The benefits of exercise extend beyond physical fitness and can have positive effects on the brain. However, an active lifestyle may be difficult for some individuals to maintain because of choice or due to age, disease, or injury. Thus, one of the enviable goals of exercise research is to identify one or many factors (such as proteins, metabolites, or enzymes) that are induced during or after exercise so it may be developed as a potential therapeutic to provide the benefits of exercise in the absence of increased physical activity. This research has typically surrounded factors that are secreted from skeletal muscle (myokines), the liver (hepatokines), and adipose tissue (adipokines) – Figure 3. Additionally, some of these factors may be released or directly induced within the brain. Not surprisingly, many of these factors regulate energy balance and glucose metabolism. Herein we focus on factors that are induced by exercise and potentially improve metabolism via action within the hypothalamus. As outlined below, the growing list of potential exercise-induced factors includes, but is not limited to: Brain-derived neurotrophic factor (BDNF), Interleukin 6 (IL-6), Ghrelin/Liver-expressed antimicrobial peptide 2 (LEAP2), N-lactoyl-phenylalanine (Lac-Phe), and Temperature.

### 3.1 Brain-derived neurotrophic factor (BDNF)

BDNF is a secreted protein that plays a crucial role in neural and synaptic development as well as synaptic plasticity [110,111]. It has also been shown to have neuroprotective properties during adverse conditions such as cerebral ischemia, hypoglycemia, and neurotoxicity [112,113]. In both rodents and humans, BDNF is expressed widely throughout the adult brain, including regions such as the olfactory bulb, cortex, basal forebrain, hippocampus, and hypothalamus [114,115]. While BDNF within the hippocampus has been linked to improvements in learning and memory or cognition [116], hypothalamic BDNF action has been linked to the regulation of energy homeostasis. In particular, deficiency of BDNF or its receptor, Tropomyosin receptor kinase B (TrkB), either globally or in the brain, has been linked to excess weight gain in rodents and humans [117,118]. Within specific hypothalamic regions such as the ARC, VMH, PVH, and DMH, BDNF deficiency or impaired signaling results in positive energy balance, leading to weight gain and/or impairment in proper responses to blood glucose levels [111,119]. Conversely, intracerebroventricular (ICV) administration of BDNF suppresses appetite, increases energy expenditure, and induces weight loss in mice [115]. These ICV administered BDNF effects can be more directly mimicked with an infusion of BDNF into the PVH and VMH of the hypothalamus [120,121]. At the cellular level, impairment of BDNF signaling in the hypothalamus leads to decreased synaptic development, maintenance, and pruning [111,119,122]. Thus, BDNF is considered a prototypical neurotrophin involved in the plasticity of hypothalamic circuits, regulating energy balance and metabolism.

Exercise increases BDNF levels in humans, regardless of the intensity (both strength and endurance) or duration (both acute and chronic) of the exercise [[123], [124], [125]]. Similarly, acute exercise increases BDNF expression in the hippocampus and hypothalamus in rodent species [126]. Interestingly, elevated levels of BDNF in the rat hippocampus may be sustained for several weeks after exercise [127]. This could be a link to the observed cognitive and metabolic improvements in physically active individuals. Some recent work suggested that exercise-induced increases in BDNF levels may be mediated by myokines such as cathepsin B and irisin [128]. It should be noted that the effects of exercise on irisin have been contentious due to debates over the specificity of reagents and potential species differences [129,130]. Nonetheless, several reports have linked irisin to improvements in cognitive function, including in individuals with Alzheimer's disease (AD), possibly involving BDNF signaling [131,132]. Additionally, prolonged exercise increases the ketone body, d-β-hydroxybutyrate (DBHB), within the liver, which can cross the blood-brain barrier and reach the mouse hippocampus [133]. Once in the hippocampus, DBHB induces BDNF expression through the inhibition of histone deacetylase (HDAC2/HDAC3) and Histone H3 acetylation [133]. Skeletal BDNF may also induce insulin secretion independent of CNS activity in rodents [134]. Importantly, there is limited knowledge regarding the possible BDNF-dependent changes in hypothalamic circuits that take place during or after exercise, and the specific mechanism responsible for inducing BDNF in response to exercise is not fully understood. However, BDNF is a potential candidate for the exercise-induced plasticity within the hypothalamus that may contribute to enduring changes in metabolism.

### 3.2 Interleukin 6 (IL-6)

IL-6, a myokine with pleiotropic functions, has diverse effects on the immune system, inflammation response, hematopoiesis, and organ development [135]. In the periphery, exercise increases IL-6 levels in human plasma, simultaneously enhancing glucose production and glucose uptake [136]. Additionally, elevated IL-6 levels have been observed in disease conditions such as cancer cachexia, where it decreases appetite, enhances energy metabolism, and increases body temperature in rodents [137,138]. Consequently, these effects result in decreased body fat mass and an increase in energy expenditure [137,138]. Conversely, mice deficient in IL-6 develop obesity in the later stages of life [139].

In addition to release from peripheral muscle tissue, IL-6 is also found within astrocytes and microglia within the CNS [140]. In rodents, the anti-obesity effect of IL-6 is partially exerted at the level of the brain, perhaps in the hypothalamus and hindbrain [139,[141], [142], [143]]. In the hypothalamus of rodents, IL-6 receptors are expressed within the ARC, DMH, VMH, PVH, and LH [143]. Neurons, including those in the hypothalamus, may also produce IL-6 in response to physical activity, a change in the strength of neuronal activity, or in response to disease/injury [140,[144], [145], [146]]. In the CNS, IL-6 has been linked to neurogenesis, altered synaptic transmission, and synaptic plasticity; potentially leading to improvements in cognition in rodents [140,145]. Chronic exercise-induced IL-6 suppresses hyperphagia mediated by overnutrition [143]. This effect involves a decrease in NPY mRNA and an increase in POMC mRNA predominantly in the arcuate nucleus of obese animals [143,145]. However, it is currently unclear if these changes in NPY and POMC levels correlate with altered cellular activity or synaptic plasticity within the hypothalamus. Intracerebroventricular administration of IL-6 also increases mitochondrial stress, an analogous response seen in POMC neurons after two weeks of moderate exercise [147]. An acute bout of exercise can also increase IL-6 mRNA levels in the VMH of the hypothalamus, where IL-6 signaling has a critical role in regulating fatty acid metabolism [148].

In addition to potential action within the hypothalamus, multiple reports demonstrate that elevated IL-6 levels (in response to a high or moderate intensity exercise) stimulate GLP-1 secretion in the periphery of both humans and rodents [149,150]. Interestingly, preproglucagon (PPG) neurons of the mouse hindbrain NTS also express excitatory IL-6 receptors [146]. Given that, in both humans and rodents, exercise increases IL-6 release in the periphery and the brain [151], elevated IL-6 levels during and after exercise may link activation of PPG neurons in the hindbrain to the beneficial effects of exercise.

### 3.3 Ghrelin

Ghrelin is a stomach-derived hormone commonly referred to as the “hunger hormone” [152,153]. Ghrelin levels rise prior to a meal and rapidly fall after a meal [152]. Accordingly, pharmacological administration of ghrelin results in an increase in food intake [154]. Ghrelin is also involved in nutritional and metabolic state-dependent regulation of blood glucose levels [155]. The ghrelin receptor, growth hormone secretagogue receptor (GHSR), is found throughout the periphery and brain in both rodents and humans [156,157]. In particular, GHSR expression is prominent in the aforementioned hypothalamic regions resulting in the ghrelin-induced direct activation of arcuate NPY/AgRP neurons and indirect inhibition of arcuate POMC neurons [158]. Additional work suggests that ghrelin and its mimetics, the growth hormone secretagogues, increase food intake and adiposity by acting (at least in part) on this circuit [159,160]. Interestingly, much of the orexigenic effect of ghrelin is diminished or completely lost in obese animals [161]. This is believed to be due, at least in part, to the rise of the GHSR antagonist/inverse agonist, Liver-expressed antimicrobial peptide 2 (LEAP2) [50,162]. LEAP2 competes with ghrelin for binding and activity of GHSR and directly counteracts many of the metabolic actions of ghrelin [50,162,163].

Exercise transiently increases ghrelin levels in plasma, and GHSRs are required for complete exercise performance in mice [37]. Several studies in humans have also found that moderate or high-intensity exercise increases ghrelin levels in both healthy, active individuals and inactive, obese individuals [[164], [165], [166]]. Interestingly, food intake after exercise is dramatically decreased in GHSR-null mice, supporting a role for ghrelin signaling in feeding after exercise [37]. Importantly, this work did not investigate the ghrelin and LEAP2-induced effects of exercise on feeding or performance via the central nervous system. However, these data raise important considerations for targeting GHSRs during and after exercise. In particular, suppressing GHSR signaling via increased LEAP2 may be a means to acquire the added benefit of exercise while limiting food intake [50]. For these observations, a thorough examination of the requirement of GHSRs as well as the interaction between ghrelin and LEAP2 in the brain is warranted.

### 3.4 Lactate and N-lactoyl-phenylalanine (Lac-Phe)

In addition to circulating peptides and neurotrophic factors, recent interest has turned to metabolites that are induced in response to increased physical activity or exercise. Lactate is a metabolite which is a valuable energy source for the brain, heart, and skeletal muscle [167]. Arterial and cerebral lactate concentrations increase in response to both low and high-intensity exercise in both rodents and humans [168,169]. Tanycytes within the third ventricle shuttle lactate to ARC POMC neurons in order to be used as an energy substrate, and this energy is necessary to sustain POMC neuronal activity. Inhibition of this lactate-POMC pathway alters energy balance [170,171]. Recent work found that an acute bout of exercise induced an increase in lactate levels and POMC expression for at least 24 h, as well as a decrease in NPY expression for 6 h in the hypothalamus of mice which was concomitant with a decrease in food intake [172]. Interestingly the exercise induced hypophagia (decreased food intake) was abolished with administration of oxamate (a LDHA inhibitor) , suggesting that central lactate could be required for exercise-induced anorexigenic effects [172]. While lactate has been linked to feeding behavior via action within the hypothalamus, it is important to note that a recent study suggested mice commonly react to an injection of Sodium l-lactate with malaise and stress, causing a natural decrease in locomotion and appetite. However, the suppression of food intake and decreased body weight in DIO mice in this study was due to the hypertonicity of the metal ion in Sodium l-lactate, rather than the lactate itself [173]. This is not necessarily a criticism of all previous studies examining the effects of lactate, as numerous studies have controlled for lactate hypertonicity and observed significant effects independent of osmolarity changes [174,175]. However, the potential influence of hypertonicity and metal ions in metabolite salts on energy metabolism should be an important consideration when determining control groups.

In addition to lactate, N-latoyl-amino acids (which are ubiquitous metabolites that are formed from lactate and amino acids) have also been suggested as a potential exerkine [176,177]. In 2015, Jansen and colleagues first discovered the molecule Lac-Phe and other N-lactoyl-amino acids as substrates of ATP-binding cassette subfamily C member 5 [176]. Notably, plasma levels of these metabolites are elevated in response to exercise in multiple species, from rodents to humans [177]. Endogenous levels of Lac-Phe correlate with exercise intensity; as high-intensity, maximal effort exercise such as running or sprinting results in higher plasma Lac-Phe levels compared to endurance and resistance training. Exercise-induced elevations in Lac-Phe are also transient across species, with levels staying elevated for at least 1-h following an exercise bout. Intraperitoneal injection of Lac-Phe increased circulating levels of the metabolite and suppressed food intake independent of locomotor activity or energy expenditure in a diet-induced obese (DIO) mouse model [117]. It should be noted that the pharmacological effect of Lac-Phe on food intake was dependent upon plasma concentrations that were 100 times higher than plasma concentrations observed after exercise. However, in support of a physiological role for Lac-Phe in this process, mice deficient for the enzyme that synthesizes Lac-Phe, cytosolic nonspecific di-peptidase 2 (CNDP2), exhibited an abrogation of the exercise-induced acute suppression of feeding. While these findings highlight a metabolite that pharmacologically and physiologically could alter energy balance in response to exercise, it is currently unclear whether this factor alters activity within the brain or, more specifically, in the hypothalamic populations highlighted in the current review. Speculatively, it is of interest that the levels of Lac-Phe remain elevated for approximately 1-h following exercise, a timeline that mirrors the suppression of ARC NPY/AgRP neuron activity following exercise. Understanding the actions of Lac-Phe in ARC NPY/AgRP neurons and other cell populations highlighted here will undoubtedly be required to better understand the cellular mechanisms behind exercise-induced metabolic responses.

### 3.5 Temperature

Core body temperature is determined by the heat equilibrium between the internal metabolic heat-production and the heat-exchange rate from the environment [178]. In general, both aerobic and anaerobic exercise is accompanied by elevations in core body temperature [179]. Exercise also increases temperature within the brain in both humans and rodents [180,181]. In particular, increases in cortical brain temperature begin within the initial phase of running (∼8 min), and the magnitude of increase in temperature is largely dependent upon running speed [180]. This effect was not limited to the cortex, as exercise-induced increases in temperature were also observed in the rat hypothalamus [182]. Separately, increasing the temperature of hypothalamic brain slices containing ARC POMC and NPY/AgRP neurons ex-vivo was sufficient to depolarize ARC POMC neurons, but not NPY/AgRP neurons [42]. The capsaicin-sensitive transient receptor potential vanilloid 1 (TRPV1) receptor antagonists and Trpv1 gene knockdown blocked the temperature-dependent depolarization of POMC neurons. Optogenetic stimulation of TRPV1 receptor-expressing POMC neurons reduced food intake, which is analogous to the acute effect observed following high-intensity exercise [42]. Moreover, knockdown of the Trpv1 gene in the ARC POMC neurons blocked the exercise-induced hypophagia [42]. These findings highlight a potential mechanism by which exercise increases temperature within the hypothalamus, thereby increasing ARC POMC activity, and consequently suppressing food intake.

It is of interest that increasing the temperature of hypothalamic brain slices ex-vivo failed to alter the activity of adjacent NPY/AgRP neurons [183]. Conversely, cold is a potent inducer of hyperphagia [184]. Recent work has demonstrated that ARC NPY/AgRP neurons are rapidly activated when mice are exposed to cold environments [185]. Moreover, cold-induced hyperphagia is abrogated when ARC NPY/AgRP neurons are inhibited [185]. Increasing the housing temperature of the mice also resulted in the reversal of the cold-induced increase in ARC NPY/AgRP activity, suggesting that elevations in temperature – depending on the magnitude – may suppress NPY/AgRP activity in-vivo [185].

It should be noted that while TRPV1 expression is localized in hypothalamic and hindbrain areas [186] and the ARC may not have the densest overall expression. Thus, one cannot exclude an indirect activation of POMC neurons in response to increased temperature in the previous report [42]. Moreover, treadmill running in rats resulted in an increase in cFos within the medial and ventromedial preoptic areas (mPOA and vmPOA), median preoptic nucleus (MnPO), PVH and supraoptic nucleus (SON), and septohypothalamic nucleus (SHy) [182]. It is unclear if these effects are a direct result of exercise, however this may suggest that exercise linked to temperature-induced changes could be a factor in the regulation of cellular activity in other intra-hypothalamic and extra-hypothalamic areas.

## 4 Conclusion

Exercise improves metabolism through numerous molecular and neuronal adaptations in both the periphery and the central nervous system (CNS) [5,6]. The melanocortin neurons in the hypothalamic ARC as well as their upstream and downstream target nuclei (e.g. the PVH, DMH and VMH) contribute to a distributed network of neurons involved in metabolism [17,19,21,25,47,49,51,70,84]. Importantly, this neuronal network is sensitive to factors produced both peripherally and within the CNS in response to exercise. While there is increasing evidence supporting the role of these factors in promoting exercise-induced metabolic adaptations, further investigation is required to better understand the regulation of metabolism in response to exercise and the specific roles of these various factors in this process. It is worth noting that not all exercise-induced factors may directly impact the hypothalamus. For example, GDF15 is a potential exercise-induced factor that may contribute to plasticity within the brain [108,109]. However, an important distinction is that the GDF15 receptor, GFRAL, is largely restricted to an area in the hindbrain. Although this does not necessarily rule out an indirect regulation of hypothalamic plasticity by GDF15, we have chosen not to discuss GDF15 in this context due to its predominantly indirect nature of regulation. In addition to these conclusions, several contextual factors (outlined below) should be considered in future studies.

First, the effects of exercise must be understood in highly heterogeneous cell populations. Within each nucleus, different cell populations can have opposing effects on energy balance and glucose metabolism. These populations may be defined by neurotransmitter and/or neuropeptide expression, as well as by how they respond to distinct interoceptive signals and food-related cues. Moreover, these cell populations may be segregated based on their projection patterns to other target nuclei, which in turn can influence metabolic outcomes. For instance, ARC, PVH, and LHA cell populations are prototypical examples of this segregation. Within the ARC, activating NPY/AgRP neurons drives feeding behavior, while activating POMC neurons suppresses feeding [31,32,55], and these effects on feeding are influenced by specific projection patterns. This was evidenced by acute activation of ARC NPY/AgRP neurons that project to the anterior subdivisions of the bed nucleus of the stria terminalis (aBNST), PVH, LHA, and paraventricular thalamic nucleus (PVT) induce feeding, while activation of those that project to the central nucleus of the amygdala (CEA) and periaqueductal grey (PAG) fail to elicit food intake [52,54]. ARC POMC neurons are also highly heterogeneous, and this differentially affects metabolism [19]. Within the PVH, acute activation of GLP-1R, oxytocin, CRH, and MC4R neurons decreases feeding and body weight, while activating PVH Sim1+/TRH+/PACAP+ neurons stimulates feeding [95,187]. Similarly, in the LHA, GABAergic neurons drive appetitive and consummatory behavior, while glutamatergic neurons in the LHA suppress feeding [188,189]. Given the innate roles of these cell populations in regulating metabolism, it is crucial for future studies to investigate the effects of exercise in a cell-specific manner to better understand the implications of exercise on metabolic outcomes.

Second, are the effects of exercise on the activity or plasticity of neurons dependent upon the time of day? Exercise induces changes in factors released into circulation or directly within the brain [5,6,37,136,177]. Metabolic responses to exercise and to these factors are variable depending on nutrient state [19], sex [190], adiposity [191], and type of exercise performed [192]. In addition, the time of day in which exercise is performed can also affect the release of these factors and the resulting metabolic changes, such as glucose production, utilization, and systemic energy expenditure [193]. The molecular mechanisms underlying these processes may also differ depending on whether exercise is performed during the active or rest phase of the day [193]. Importantly, the expression level of neuropeptides and neuronal activity in the brain, including hypothalamic nuclei, depends upon diurnal variation in order to reflect different physiological needs according to circadian rhythm [19,[194], [195], [196]]. While not entirely clear, the diurnal variability of metabolically relevant neural circuits likely contributes to differing metabolic outcomes of exercise. These findings highlight the importance of investigating the temporally-dependent effects of exercise on the plasticity of brain circuits and their associations with metabolic improvements.

Third, what circuits in the brain are required for the exercise-induced beneficial effects on metabolism, and how are they connected? The current review highlights an emerging field of research with the goal of identifying circuit nodes (nuclei and specific cell populations) in the brain that are modified in response to exercise and/or contribute to exercise performance. Using molecular genetics and the ability to manipulate the activity of neurons in a cell-specific manner, previous studies have identified metabolically-relevant circuits in the brain that may be involved in regulating metabolism in response to exercise. However, it is still unclear whether all metabolically-relevant nuclei/cell populations are modified in response to exercise and how these circuits are interconnected. Additionally, it is currently unknown if these circuits are required for exercise-induced changes in metabolism or exercise performance. Similar to the recent advances in neuroscience research that has accelerated our understanding of the brain's involvement in energy balance and glucose control, a combinatorial approach of optical imaging, electrical recordings, and circuit mapping tools will be needed to parse these important questions.

Another key aspect to consider is the duration of exercise-induced effects on individual neurons and neural circuits. Numerous studies investigating the effects of exercise in the periphery and the CNS focus on a perceived, arbitrary time-point, which may not capture the dynamic nature of exercise-induced changes. Moreover, the changes in neuron activity observed in response to exercise may be transient or sustained for hours to days after an exercise bout. This may be less surprising given that exercise induces a rapid increase in metabolic rate and glucose uptake that is sustained post-exercise across multiple species [197]. Such a sustained change in activity may also be an adaptation to exercise in order to ensure future exercise performance. A common principle in exercise training revolves around training reversibility and supports the phrase “Use it or Lose it”. That is, while regularly performed aerobic exercise results in significant improvements in metabolism, cessation of exercise training results in decline in various metabolic parameters. Neural plasticity is likely to play a role in these adaptations, and it is important to understand the onset and reversal of exercise-induced plasticity in order to better understand exercise-induced remodeling within the brain. Future studies should consider the time course of exercise-induced changes in neuronal activity and how these changes contribute to the sustained metabolic benefits of exercise.

Finally, studies on the adaptive capacity of metabolically relevant neurons or brain circuits commonly center on studying neuroplasticity in lean subjects. While these studies provide crucial baseline insights into potential cellular mechanisms contributing to metabolic dysfunction, it is equally important to investigate whether and how these circuits can adapt in the context of disease states. This is particularly important for understanding the effects of exercise on metabolic dysfunction and whether lifestyle interventions, such as increased physical activity or exercise, can induce similar plasticity changes as observed in healthy individuals.

Proper consideration of these contextual factors will significantly contribute to our understanding of the effects of exercise on metabolism and may explain the inter-individual variability in response to exercise. Ultimately, investigating these factors will be crucial for elucidating a more comprehensive understanding of the mechanisms underlying exercise-induced metabolic adaptations.

## Declaration of competing interest

The authors declare that they have no known competing financial interests or personal relationships that could have appeared to influence the work reported in this paper.

## Data Availability

No data was used for the research described in the article.

## References

[1] Nystoriak M.A., Bhatnagar A. (2018). Cardiovascular effects and benefits of exercise. Front Cardiovasc Med.

[2] O'Callaghan R.M., Ohle R., Kelly A.M. (2007). The effects of forced exercise on hippocampal plasticity in the rat: a comparison of LTP, spatial- and non-spatial learning. Behav Brain Res.

[3] Griffin E.W., Bechara R.G., Birch A.M., Kelly A.M. (2009). Exercise enhances hippocampal-dependent learning in the rat: evidence for a BDNF-related mechanism. Hippocampus.

[4] Gomez-Pinilla F., Hillman C. (2013). The influence of exercise on cognitive abilities. Compr Physiol.

[5] Egan B., Zierath J.R. (2013). Exercise metabolism and the molecular regulation of skeletal muscle adaptation. Cell Metab.

[6] Goodyear L.J., Kahn B.B. (1998). Exercise, glucose transport, and insulin sensitivity. Annu Rev Med.

[7] Green D.J., Maiorana A., O'Driscoll G., Taylor R. (2004). Effect of exercise training on endothelium-derived nitric oxide function in humans. J Physiol Lond.

[8] Hambrecht R., Adams V., Erbs S., Linke A., Krankel N., Shu Y. (2003). Regular physical activity improves endothelial function in patients with coronary artery disease by increasing phosphorylation of endothelial nitric oxide synthase. Circulation.

[9] Horowitz A.M., Fan X.L., Bieri G., Smith L.K., Sanchez-Diaz C.I., Schroer A.B. (2020). Blood factors transfer beneficial effects of exercise on neurogenesis and cognition to the aged brain. Science.

[10] Colcombe S., Kramer A.F. (2003). Fitness effects on the cognitive function of older adults: a meta-analytic study. Psychol Sci.

[11] Coggan A.R., Swanson S.C., Mendenhall L.A., Habash D.L., Kien C.L. (1995). Effect of endurance training on hepatic glycogenolysis and gluconeogenesis during prolonged exercise in men. Am J Physiol.

[12] DeFronzo R.A., Sherwin R.S., Kraemer N. (1987). Effect of physical training on insulin action in obesity. Diabetes.

[13] Broom D.R., Miyashita M., Wasse L.K., Pulsford R., King J.A., Thackray A.E. (2017). Acute effect of exercise intensity and duration on acylated ghrelin and hunger in men. J Endocrinol.

[14] King J.A., Wasse L.K., Stensel D.J. (2013). Acute exercise increases feeding latency in healthy normal weight young males but does not alter energy intake. Appetite.

[15] Douglas J.A., King J.A., Clayton D.J., Jackson A.P., Sargeant J.A., Thackray A.E. (2017). Acute effects of exercise on appetite, ad libitum energy intake and appetite-regulatory hormones in lean and overweight/obese men and women. Int J Obes Lond.

[16] Morgan J.A., Corrigan F., Baune B.T. (2015). Effects of physical exercise on central nervous system functions: a review of brain region specific adaptations. J Mol Psychiatr.

[17] Alcantara I.C., Tapia A.P.M., Aponte Y., Krashes M.J. (2022). Acts of appetite: neural circuits governing the appetitive, consummatory, and terminating phases of feeding. Nat Metab.

[18] Williams K.W., Elmquist J.K. (2012). From neuroanatomy to behavior: central integration of peripheral signals regulating feeding behavior. Nat Neurosci.

[19] Lieu L., Chau D., Afrin S., Dong Y., Alhadeff A.L., Betley J.N. (2020). Effects of metabolic state on the regulation of melanocortin circuits. Physiol Behav.

[20] Alhadeff A.L. (2021). Monitoring in vivo neural activity to understand gut-brain signaling. Endocrinology.

[21] Myers M.G., Olson D.P. (2012). Central nervous system control of metabolism. Nature.

[22] Stuber G.D., Wise R.A. (2016). Lateral hypothalamic circuits for feeding and reward. Nat Neurosci.

[23] van Iersel L., Brokke K.E., Adan R.A.H., Bulthuis L.C.M., van den Akker E.L.T., van Santen H.M. (2019). Pathophysiology and individualized treatment of hypothalamic obesity following craniopharyngioma and other suprasellar tumors: a systematic review. Endocr Rev.

[24] Williams K.W., Scott M.M., Elmquist J.K. (2011). Modulation of the central melanocortin system by leptin, insulin, and serotonin: co-ordinated actions in a dispersed neuronal network. Eur J Pharmacol.

[25] Alonge K.M., D'Alessio D.A., Schwartz M.W. (2021). Brain control of blood glucose levels: implications for the pathogenesis of type 2 diabetes. Diabetologia.

[26] Oomura Y., Ooyama H., Sugimori M., Nakamura T., Yamada Y. (1974). Glucose inhibition of the glucose-sensitive neurone in the rat lateral hypothalamus. Nature.

[27] Fioramonti X., Lorsignol A., Taupignon A., Penicaud L. (2004). A new ATP-sensitive K+ channel-independent mechanism is involved in glucose-excited neurons of mouse arcuate nucleus. Diabetes.

[28] Kang L., Routh V.H., Kuzhikandathil E.V., Gaspers L.D., Levin B.E. (2004). Physiological and molecular characteristics of rat hypothalamic ventromedial nucleus glucosensing neurons. Diabetes.

[29] Meek T.H., Nelson J.T., Matsen M.E., Dorfman M.D., Guyenet S.J., Damian V. (2016). Functional identification of a neurocircuit regulating blood glucose. Proc Natl Acad Sci U S A.

[30] Stanley S.A., Kelly L., Latcha K.N., Schmidt S.F., Yu X.F., Nectow A.R. (2016). Bidirectional electromagnetic control of the hypothalamus regulates feeding and metabolism. Nature.

[31] Aponte Y., Atasoy D., Sternson S.M. (2011). AGRP neurons are sufficient to orchestrate feeding behavior rapidly and without training. Nat Neurosci.

[32] Atasoy D., Betley J.N., Su H.H., Sternson S.M. (2012). Deconstruction of a neural circuit for hunger. Nature.

[33] Fenselau H., Campbell J.N., Verstegen A.M., Madara J.C., Xu J., Shah B.P. (2017). A rapidly acting glutamatergic ARC-->PVH satiety circuit postsynaptically regulated by alpha-MSH. Nat Neurosci.

[34] Barbano M.F., Wang H.L., Morales M., Wise R.A. (2016). Feeding and reward are differentially induced by activating GABAergic lateral hypothalamic projections to VTA. J Neurosci.

[35] Cavalcanti-de-Albuquerque J.P., Bober J., Zimmer M.R., Dietrich M.O. (2019). Regulation of substrate utilization and adiposity by Agrp neurons. Nat Commun.

[36] Fujikawa T., Castorena C.M., Pearson M., Kusminski C.M., Ahmed N., Battiprolu P.K. (2016). SF-1 expression in the hypothalamus is required for beneficial metabolic effects of exercise. Elife.

[37] Mani B.K., Castorena C.M., Osborne-Lawrence S., Vijayaraghavan P., Metzger N.P., Elmquist J.K. (2018). Ghrelin mediates exercise endurance and the feeding response post-exercise. Mol Metab.

[38] Oberlin D.J., Mikus C.R., Kearney M.L., Hinton P.S., Manrique C., Leidy H.J. (2014). One bout of exercise alters free-living postprandial glycemia in type 2 diabetes. Med Sci Sports Exerc.

[39] Manders R.J., Van Dijk J.W., van Loon L.J. (2010). Low-intensity exercise reduces the prevalence of hyperglycemia in type 2 diabetes. Med Sci Sports Exerc.

[40] Mikines K.J., Sonne B., Farrell P.A., Tronier B., Galbo H. (1988). Effect of physical exercise on sensitivity and responsiveness to insulin in humans. Am J Physiol.

[41] Van Dijk J.W., Manders R.J., Canfora E.E., Mechelen W.V., Hartgens F., Stehouwer C.D. (2013). Exercise and 24-h glycemic control: equal effects for all type 2 diabetes patients?. Med Sci Sports Exerc.

[42] Jeong J.H., Lee D.K., Liu S.M., Chua S.C., Schwartz G.J., Jo Y.H. (2018). Activation of temperature-sensitive TRPV1-like receptors in ARC POMC neurons reduces food intake. PLoS Biol.

[43] He Z., Gao Y., Alhadeff A.L., Castorena C.M., Huang Y., Lieu L. (2018). Cellular and synaptic reorganization of arcuate NPY/AgRP and POMC neurons after exercise. Mol Metab.

[44] Dong Y., Carty J., Goldstein N., He Z., Hwang E., Chau D. (2021). Time and metabolic state-dependent effects of GLP-1R agonists on NPY/AgRP and POMC neuronal activity in vivo. Mol Metab.

[45] Anderson E.J., Cakir I., Carrington S.J., Cone R.D., Ghamari-Langroudi M., Gillyard T. (2016). 60 years of POMC: regulation of feeding and energy homeostasis by alpha-MSH. J Mol Endocrinol.

[46] Schwartz M.W., Porte D. (2005). Diabetes, obesity, and the brain. Science.

[47] Contreras C., Nogueiras R., Dieguez C., Rahmouni K., Lopez M. (2017). Traveling from the hypothalamus to the adipose tissue: the thermogenic pathway. Redox Biol.

[48] Caron A., Lee S., Elmquist J.K., Gautron L. (2018). Leptin and brain-adipose crosstalks. Nat Rev Neurosci.

[49] Gautron L., Elmquist J.K. (2011). Sixteen years and counting: an update on leptin in energy balance. J Clin Invest.

[50] Gupta D., Ogden S.B., Shankar K., Varshney S., Zigman J.M. (2021). A LEAP 2 conclusions? Targeting the ghrelin system to treat obesity and diabetes. Mol Metab.

[51] Gautron L., Elmquist J.K., Williams K.W. (2015). Neural control of energy balance: translating circuits to therapies. Cell.

[52] Betley J.N., Cao Z.F., Ritola K.D., Sternson S.M. (2013). Parallel, redundant circuit organization for homeostatic control of feeding behavior. Cell.

[53] Krashes M.J., Koda S., Ye C., Rogan S.C., Adams A.C., Cusher D.S. (2011). Rapid, reversible activation of AgRP neurons drives feeding behavior in mice. J Clin Invest.

[54] Steculorum S.M., Ruud J., Karakasilioti I., Backes H., Engstrom Ruud L., Timper K. (2016). AgRP neurons control systemic insulin sensitivity via myostatin expression in brown adipose tissue. Cell.

[55] Krashes M.J., Shah B.P., Koda S., Lowell B.B. (2013). Rapid versus delayed stimulation of feeding by the endogenously released AgRP neuron mediators GABA, NPY, and AgRP. Cell Metab.

[56] Uner A.G., Kecik O., Quaresma P.G.F., De Araujo T.M., Lee H., Li W. (2019). Role of POMC and AgRP neuronal activities on glycaemia in mice. Sci Rep.

[57] Hruby V.J., Lu D., Sharma S.D., Castrucci A.L., Kesterson R.A., al-Obeidi F.A. (1995). Cyclic lactam alpha-melanotropin analogues of Ac-Nle4-cyclo[Asp5, D-Phe7,Lys10] alpha-melanocyte-stimulating hormone-(4-10)-NH2 with bulky aromatic amino acids at position 7 show high antagonist potency and selectivity at specific melanocortin receptors. J Med Chem.

[58] Pinto S., Roseberry A.G., Liu H., Diano S., Shanabrough M., Cai X. (2004). Rapid rewiring of arcuate nucleus feeding circuits by leptin. Science.

[59] Berrios J., Li C., Madara J.C., Garfield A.S., Steger J.S., Krashes M.J. (2021). Food cue regulation of AGRP hunger neurons guides learning. Nature.

[60] Chen Y.M., Lin Y.C., Kuo T.W., Knight Z.A. (2015). Sensory detection of food rapidly modulates arcuate feeding circuits. Cell.

[61] Goldstein N., McKnight A.D., Carty J.R.E., Arnold M., Betley J.N., Alhadeff A.L. (2021). Hypothalamic detection of macronutrients via multiple gut-brain pathways. Cell Metab.

[62] Su Z., Alhadeff A.L., Betley J.N. (2017). Nutritive, post-ingestive signals are the primary regulators of AgRP neuron activity. Cell Rep.

[63] Miletta M.C., Iyilikci O., Shanabrough M., Sestan-Pesa M., Cammisa A., Zeiss C.J. (2020). AgRP neurons control compulsive exercise and survival in an activity-based anorexia model. Nat Metab.

[64] Romijn J.A., Coyle E.F., Sidossis L.S., Gastaldelli A., Horowitz J.F., Endert E. (1993). Regulation of endogenous fat and carbohydrate metabolism in relation to exercise intensity and duration. Am J Physiol.

[65] Bergman B.C., Brooks G.A. (1999). Respiratory gas-exchange ratios during graded exercise in fed and fasted trained and untrained men. J Appl Physiol (1985).

[66] Xu Y., O'Brien W.G., Lee C.C., Myers M.G., Tong Q. (2012). Role of GABA release from leptin receptor-expressing neurons in body weight regulation. Endocrinology.

[67] Campbell J.N., Macosko E.Z., Fenselau H., Pers T.H., Lyubetskaya A., Tenen D. (2017). A molecular census of arcuate hypothalamus and median eminence cell types. Nat Neurosci.

[68] Kim E.R., Wu Z., Sun H., Xu Y., Mangieri L.R., Xu Y. (2015). Hypothalamic non-AgRP, non-POMC GABAergic neurons are required for postweaning feeding and NPY hyperphagia. J Neurosci.

[69] Suyama S., Yada T. (2018). New insight into GABAergic neurons in the hypothalamic feeding regulation. J Physiol Sci.

[70] Choi Y.H., Fujikawa T., Lee J., Reuter A., Kim K.W. (2013). Revisiting the ventral medial nucleus of the hypothalamus: the roles of SF-1 neurons in energy homeostasis. Front Neurosci.

[71] Haque M.S., Minokoshi Y., Hamai M., Iwai M., Horiuchi M., Shimazu T. (1999). Role of the sympathetic nervous system and insulin in enhancing glucose uptake in peripheral tissues after intrahypothalamic injection of leptin in rats. Diabetes.

[72] Bamshad M., Song C.K., Bartness T.J. (1999). CNS origins of the sympathetic nervous system outflow to brown adipose tissue. Am J Physiol.

[73] Coutinho E.A., Okamoto S., Ishikawa A.W., Yokota S., Wada N., Hirabayashi T. (2017). Activation of SF1 neurons in the ventromedial hypothalamus by DREADD technology increases insulin sensitivity in peripheral tissues. Diabetes.

[74] Scheurink A.J., Steffens A.B., Benthem L. (1988). Central and peripheral adrenoceptors affect glucose, free fatty acids, and insulin in exercising rats. Am J Physiol.

[75] Miyaki T., Fujikawa T., Kitaoka R., Hirano N., Matsumura S., Fushiki T. (2011). Noradrenergic projections to the ventromedial hypothalamus regulate fat metabolism during endurance exercise. Neuroscience.

[76] Fujikawa T., Castorena C.M., Lee S., Elmquist J.K. (2017). The hypothalamic regulation of metabolic adaptations to exercise. J Neuroendocrinol.

[77] Krause W.C., Rodriguez R., Gegenhuber B., Matharu N., Rodriguez A.N., Padilla-Roger A.M. (2021). Oestrogen engages brain MC4R signalling to drive physical activity in female mice. Nature.

[78] Sternson S.M., Shepherd G.M.G., Friedman J.M. (2005). Topographic mapping of VMH -> arcuate nucleus microcircuits and their reorganization by fasting. Nat Neurosci.

[79] Bellinger L.L., Bernardis L.L. (2002). The dorsomedial hypothalamic nucleus and its role in ingestive behavior and body weight regulation: lessons learned from lesioning studies. Physiol Behav.

[80] Zaretskaia M.V., Zaretsky D.V., Shekhar A., DiMicco J.A. (2002). Chemical stimulation of the dorsomedial hypothalamus evokes non-shivering thermogenesis in anesthetized rats. Brain Res.

[81] Cao W.H., Fan W., Morrison S.F. (2004). Medullary pathways mediating specific sympathetic responses to activation of dorsomedial hypothalamus. Neuroscience.

[82] Maejima Y., Yokota S., Shimizu M., Horita S., Kobayashi D., Hazama A. (2021). The deletion of glucagon-like peptide-1 receptors expressing neurons in the dorsomedial hypothalamic nucleus disrupts the diurnal feeding pattern and induces hyperphagia and obesity. Nutr Metab Lond.

[83] Lee S.J., Sanchez-Watts G., Krieger J.P., Pignalosa A., Norell P.N., Cortella A. (2018). Loss of dorsomedial hypothalamic GLP-1 signaling reduces BAT thermogenesis and increases adiposity. Mol Metab.

[84] Enriori P.J., Sinnayah P., Simonds S.E., Garcia Rudaz C., Cowley M.A. (2011). Leptin action in the dorsomedial hypothalamus increases sympathetic tone to brown adipose tissue in spite of systemic leptin resistance. J Neurosci.

[85] Garfield A.S., Shah B.P., Burgess C.R., Li M.M., Li C., Steger J.S. (2016). Dynamic GABAergic afferent modulation of AgRP neurons. Nat Neurosci.

[86] Zhang N., Yang L., Guo L., Bi S. (2018). Activation of dorsomedial hypothalamic neurons promotes physical activity and decreases food intake and body weight in Zucker fatty rats. Front Mol Neurosci.

[87] Zheng F., Kim Y.J., Moran T.H., Li H., Bi S. (2016). Central transthyretin acts to decrease food intake and body weight. Sci Rep.

[88] Zaretsky D.V., Kline H., Zaretskaia M.V., Brown M.B., Durant P.J., Alves N.J. (2018). Disinhibiting neurons in the dorsomedial hypothalamus delays the onset of exertional fatigue and exhaustion in rats exercising in a warm environment. Brain Res.

[89] Gold R.M., Jones A.P., Sawchenko P.E., Kapatos G. (1977). Paraventricular area - critical focus of a longitudinal neurocircuitry mediating food-intake. Physiol Behav.

[90] Leibowitz S.F., Hammer N.J., Chang K. (1981). Hypothalamic paraventricular nucleus lesions produce overeating and obesity in the rat. Physiol Behav.

[91] Garfield A.S., Li C., Madara J.C., Shah B.P., Webber E., Steger J.S. (2015). A neural basis for melanocortin-4 receptor-regulated appetite. Nat Neurosci.

[92] Farooqi I.S., Keogh J.M., Yeo G.S.H., Lank E.J., Cheetham T., O'Rahilly S. (2003). Clinical spectrum of obesity and mutations in the melanocortin 4 receptor gene. N Engl J Med.

[93] Krude H., Biebermann H., Schnabel D., Tansek M.Z., Theunissen P., Mullis P.E. (2003). Obesity due to proopiomelanocortin deficiency: three new cases and treatment trials with thyroid hormone and ACTH4-10. J Clin Endocrinol Metab.

[94] Sutton A.K., Pei H.J., Burnett K.H., Myers M.G., Rhodes C.J., Olson D.P. (2014). Control of food intake and energy expenditure by Nos1 neurons of the paraventricular hypothalamus. J Neurosci.

[95] Li C., Navarrete J., Liang-Guallpa J., Lu C.X., Funderburk S.C., Chang R.B. (2019). Defined paraventricular hypothalamic populations exhibit differential responses to food contingent on caloric state. Cell Metabol.

[96] Ryan P.J., Ross S.I., Campos C.A., Derkach V.A., Palmiter R.D. (2017). Oxytocin-receptor-expressing neurons in the parabrachial nucleus regulate fluid intake. Nat Neurosci.

[97] Wu Z.F., Xu Y.Z., Zhu Y.M., Sutton A.K., Zhao R.J., Lowell B.B. (2012). An obligate role of oxytocin neurons in diet induced energy expenditure. PLoS One.

[98] McMahon L.R., Wellman P.J. (1997). Decreased intake of a liquid diet in nonfood-deprived rats following intra-PVN injections of GLP-1 (7-36) amide. Pharmacol Biochem Behav.

[99] Krashes M.J., Shah B.P., Madara J.C., Olson D.P., Strochlic D.E., Garfield A.S. (2014). An excitatory paraventricular nucleus to AgRP neuron circuit that drives hunger. Nature.

[100] Doslikova B., Tchir D., McKinty A., Zhu X., Marks D.L., Baracos V.E. (2019). Convergent neuronal projections from paraventricular nucleus, parabrachial nucleus, and brainstem onto gastrocnemius muscle, white and brown adipose tissue in male rats. J Comp Neurol.

[101] Hollis J.H., Lightman S.L., Lowry C.A. (2004). Integration of systemic and visceral sensory information by medullary catecholaminergic systems during peripheral inflammation. Ann N Y Acad Sci.

[102] Saper C.B. (2002). The central autonomic nervous system: conscious visceral perception and autonomic pattern generation. Annu Rev Neurosci.

[103] Duan Y.F., Kopin I.J., Goldstein D.S. (1999). Stimulation of the paraventricular nucleus modulates firing of neurons in the nucleus of the solitary tract. Am J Physiol.

[104] Bunner W., Landry T., Laing B.T., Li P., Rao Z., Yuan Y. (2020). ARC(AgRP/NPY) neuron activity is required for acute exercise-induced food intake in un-trained mice. Front Physiol.

[105] Jackson K., Silva H.M.V., Zhang W.F., Michelini L.C., Stern J.E. (2005). Exercise training differentially affects intrinsic excitability of autonomic and neuroendocrine neurons in the hypothalamic paraventricular nucleus. J Neurophysiol.

[106] Michelini L.C., Stern J.E. (2009). Exercise-induced neuronal plasticity in central autonomic networks: role in cardiovascular control. Exp Physiol.

[107] Michelini L.C. (2007). Differential effects of vasopressinergic and oxytocinergic pre-autonomic neurons on circulatory control: reflex mechanisms and changes during exercise. Clin Exp Pharmacol Physiol.

[108] Hsu J.Y., Crawley S., Chen M., Ayupova D.A., Lindhout D.A., Higbee J. (2017). Non-homeostatic body weight regulation through a brainstem-restricted receptor for GDF15. Nature.

[109] Kleinert M., Clemmensen C., Sjoberg K.A., Carl C.S., Jeppesen J.F., Wojtaszewski J.F.P. (2018). Exercise increases circulating GDF15 in humans. Mol Metab.

[110] Binder D.K., Scharfman H.E. (2004). Brain-derived neurotrophic factor. Growth Factors.

[111] Xu B., Xie X. (2016). Neurotrophic factor control of satiety and body weight. Nat Rev Neurosci.

[112] Scalzo P., Kummer A., Bretas T.L., Cardoso F., Teixeira A.L. (2010). Serum levels of brain-derived neurotrophic factor correlate with motor impairment in Parkinson's disease. J Neurol.

[113] Sohrabji F., Lewis D.K. (2006). Estrogen-BDNF interactions: implications for neurodegenerative diseases. Front Neuroendocrinol.

[114] Murer M.G., Yan Q., Raisman-Vozari R. (2001). Brain-derived neurotrophic factor in the control human brain, and in Alzheimer's disease and Parkinson's disease. Prog Neurobiol.

[115] Kernie S.G., Liebl D.J., Parada L.F. (2000). BDNF regulates eating behavior and locomotor activity in mice. EMBO J.

[116] Cunha C., Brambilla R., Thomas K.L. (2010). A simple role for BDNF in learning and memory?. Front Mol Neurosci.

[117] Gray J., Yeo G.S., Cox J.J., Morton J., Adlam A.L., Keogh J.M. (2006). Hyperphagia, severe obesity, impaired cognitive function, and hyperactivity associated with functional loss of one copy of the brain-derived neurotrophic factor (BDNF) gene. Diabetes.

[118] Yeo G.S., Connie Hung C.C., Rochford J., Keogh J., Gray J., Sivaramakrishnan S. (2004). A de novo mutation affecting human TrkB associated with severe obesity and developmental delay. Nat Neurosci.

[119] Kamitakahara A., Xu B.J., Simerly R. (2016). Ventromedial hypothalamic expression of Bdnf is required to establish normal patterns of afferent GABAergic connectivity and responses to hypoglycemia. Mol Metabol.

[120] Wang C., Bomberg E., Billington C., Levine A., Kotz C.M. (2007). Brain-derived neurotrophic factor in the hypothalamic paraventricular nucleus reduces energy intake. Am J Physiol Regul Integr Comp Physiol.

[121] Wang C., Bomberg E., Levine A., Billington C., Kotz C.M. (2007). Brain-derived neurotrophic factor in the ventromedial nucleus of the hypothalamus reduces energy intake. Am J Physiol Regul Integr Comp Physiol.

[122] Rios M. (2013). BDNF and the central control of feeding: accidental bystander or essential player?. Trends Neurosci.

[123] Arazi H., Babaei P., Moghimi M., Asadi A. (2021). Acute effects of strength and endurance exercise on serum BDNF and IGF-1 levels in older men. BMC Geriatr.

[124] De la Rosa A., Solana E., Corpas R., Bartres-Faz D., Pallas M., Vina J. (2019). Long-term exercise training improves memory in middle-aged men and modulates peripheral levels of BDNF and Cathepsin B. Sci Rep.

[125] Hung C.L., Tseng J.W., Chao H.H., Hung T.M., Wang H.S. (2018). Effect of acute exercise mode on serum brain-derived neurotrophic factor (BDNF) and task switching performance. J Clin Med.

[126] Takimoto M., Hamada T. (2014). Acute exercise increases brain region-specific expression of MCT1, MCT2, MCT4, GLUT1, and COX IV proteins. J Appl Physiol.

[127] Russo-Neustadt A., Beard R.C., Cotman C.W. (1999). Exercise, antidepressant medications, and enhanced brain derived neurotrophic factor expression. Neuropsychopharmacology.

[128] Liu P., Zou D., Yi L., Chen M.L., Gao Y.X., Zhou R. (2015). Quercetin ameliorates hypobaric hypoxia-induced memory impairment through mitochondrial and neuron function adaptation via the PGC-1 alpha pathway. Restor Neurol Neurosci.

[129] Bostrom P., Wu J., Jedrychowski M.P., Korde A., Ye L., Lo J.C. (2012). Is irisin a human exercise gene? Reply. Nature.

[130] Albrecht E., Norheim F., Thiede B., Holen T., Ohashi T., Schering L. (2015). Irisin - a myth rather than an exercise-inducible myokine. Sci Rep.

[131] Islam M.R., Valaris S., Young M.F., Haley E.B., Luo R.H., Bond S.F. (2021). Exercise hormone irisin is a critical regulator of cognitive function (vol. 3, pg 1058, 2021). Nat Metab.

[132] Lourenco M.V., Ribeiro F.C., Sudo F.K., Drummond C., Assuncao N., Vanderborght B. (2020). Cerebrospinal fluid irisin correlates with amyloid-beta, BDNF, and cognition in Alzheimer's disease. Alzheimers Dement Amst.

[133] Sleiman S.F., Henry J., Al-Haddad R., El Hayek L., Abou Haidar E., Stringer T. (2016). Exercise promotes the expression of brain derived neurotrophic factor (BDNF) through the action of the ketone body beta-hydroxybutyrate. Elife.

[134] Fulgenzi G., Hong Z., Tomassoni-Ardori F., Barella L.F., Becker J., Barrick C. (2020). Novel metabolic role for BDNF in pancreatic beta-cell insulin secretion. Nat Commun.

[135] Hirano T. (1998). Interleukin 6 and its receptor: ten years later. Int Rev Immunol.

[136] Febbraio M.A., Hiscock N., Sacchetti M., Fischer C.P., Pedersen B.K. (2004). Interleukin-6 is a novel factor mediating glucose homeostasis during skeletal muscle contraction. Diabetes.

[137] Cahlin C., Korner A., Axelsson H., Wang W., Lundholm K., Svanberg E. (2000). Experimental cancer cachexia: the role of host-derived cytokines interleukin (IL)-6, IL-12, interferon-gamma, and tumor necrosis factor alpha evaluated in gene knockout, tumor-bearing mice on C57 Bl background and eicosanoid-dependent cachexia. Cancer Res.

[138] Strassmann G., Fong M., Kenney J.S., Jacob C.O. (1992). Evidence for the involvement of interleukin 6 in experimental cancer cachexia. J Clin Invest.

[139] Wallenius V., Wallenius K., Ahren B., Rudling M., Carlsten H., Dickson S.L. (2002). Interleukin-6-deficient mice develop mature-onset obesity. Nat Med.

[140] Gruol D.L. (2015). IL-6 regulation of synaptic function in the CNS. Neuropharmacology.

[141] Li G., Klein R.L., Matheny M., King M.A., Meyer E.M., Scarpace P.J. (2002). Induction of uncoupling protein 1 by central interleukin-6 gene delivery is dependent on sympathetic innervation of brown adipose tissue and underlies one mechanism of body weight reduction in rats. Neuroscience.

[142] Wallenius K., Wallenius V., Sunter D., Dickson S.L., Jansson J.O. (2002). Intracerebroventricular interleukin-6 treatment decreases body fat in rats. Biochem Biophys Res Commun.

[143] Ropelle E.R., Flores M.B., Cintra D.E., Rocha G.Z., Pauli J.R., Morari J. (2010). IL-6 and IL-10 anti-inflammatory activity links exercise to hypothalamic insulin and leptin sensitivity through IKKbeta and ER stress inhibition. PLoS Biol.

[144] Hans V.H., Kossmann T., Lenzlinger P.M., Probstmeier R., Imhof H.G., Trentz O. (1999). Experimental axonal injury triggers interleukin-6 mRNA, protein synthesis and release into cerebrospinal fluid. J Cereb Blood Flow Metab.

[145] Bobbo V.C., Engel D.F., Jara C.P., Mendes N.F., Haddad-Tovolli R., Prado T.P. (2021). Interleukin-6 actions in the hypothalamus protects against obesity and is involved in the regulation of neurogenesis. J Neuroinflammation.

[146] Anesten F., Holt M.K., Schele E., Palsdottir V., Reimann F., Gribble F.M. (2016). Preproglucagon neurons in the hindbrain have IL-6 receptor-alpha and show Ca2+ influx in response to IL-6. Am J Physiol Regul Integr Comp Physiol.

[147] Kang G.M., Min S.H., Lee C.H., Kim J.Y., Lim H.S., Choi M.J. (2021). Mitohormesis in hypothalamic POMC neurons mediates regular exercise-induced high-turnover metabolism. Cell Metab.

[148] Katashima C.K., de Oliveira Micheletti T., Braga R.R., Gaspar R.S., Goeminne L.J.E., Moura-Assis A. (2022). Evidence for a neuromuscular circuit involving hypothalamic interleukin-6 in the control of skeletal muscle metabolism. Sci Adv.

[149] Ellingsgaard H., Hauselmann I., Schuler B., Habib A.M., Baggio L.L., Meier D.T. (2011). Interleukin-6 enhances insulin secretion by increasing glucagon-like peptide-1 secretion from L cells and alpha cells. Nat Med.

[150] Islam H., Townsend L.K., McKie G.L., Medeiros P.J., Gurd B.J., Hazell T.J. (2017). Potential involvement of lactate and interleukin-6 in the appetite-regulatory hormonal response to an acute exercise bout. J Appl Physiol.

[151] Nybo L., Nielsen B., Pedersen B.K., Moller K., Secher N.H. (2002). Interleukin-6 release from the human brain during prolonged exercise. J Physiol.

[152] Castaneda T.R., Tong J., Datta R., Culler M., Tschop M.H. (2010). Ghrelin in the regulation of body weight and metabolism. Front Neuroendocrinol.

[153] Asakawa A., Inui A., Kaga T., Yuzuriha H., Nagata T., Ueno N. (2001). Ghrelin is an appetite-stimulatory signal from stomach with structural resemblance to motilin. Gastroenterology.

[154] Egecioglu E., Jerlhag E., Salome N., Skibicka K.P., Haage D., Bohlooly-Y M. (2010). Ghrelin increases intake of rewarding food in rodents. Addict Biol.

[155] Mani B.K., Shankar K., Zigman J.M. (2019). Ghrelin's relationship to blood glucose. Endocrinology.

[156] Howard A.D., Feighner S.D., Cully D.F., Arena J.P., Liberator P.A., Rosenblum C.I. (1996). A receptor in pituitary and hypothalamus that functions in growth hormone release. Science.

[157] Abizaid A., Hougland J.L. (2020). Ghrelin signaling: GOAT and GHS-r1a take a LEAP in complexity. Trends Endocrinol Metabol.

[158] Cowley M.A., Cone R.D., Enriori P., Louiselle I., Williams S.M., Evans A.E. (2003). Electrophysiological actions of peripheral hormones on melanocortin neurons. Ann N Y Acad Sci.

[159] Zhang J.V., Ren P.G., Avsian-Kretchmer O., Luo C.W., Rauch R., Klein C. (2005). Obestatin, a peptide encoded by the ghrelin gene, opposes ghrelin's effects on food intake. Science.

[160] Tschop M., Smiley D.L., Heiman M.L. (2000). Ghrelin induces adiposity in rodents. Nature.

[161] Briggs D.I., Enriori P.J., Lemus M.B., Cowley M.A., Andrews Z.B. (2010). Diet-induced obesity causes ghrelin resistance in arcuate NPY/AgRP neurons. Endocrinology.

[162] Ge X., Yang H., Bednarek M.A., Galon-Tilleman H., Chen P., Chen M. (2018). LEAP2 is an endogenous antagonist of the ghrelin receptor. Cell Metab.

[163] Mani B.K., Puzziferri N., He Z., Rodriguez J.A., Osborne-Lawrence S., Metzger N.P. (2019). LEAP2 changes with body mass and food intake in humans and mice. J Clin Invest.

[164] Crabtree D.R., Blannin A.K. (2015). Effects of exercise in the cold on Ghrelin, PYY, and food intake in overweight adults. Med Sci Sports Exerc.

[165] Saghebjoo M., Hedayati M., Fahimi Y., Ilbeigi S. (2013). Plasma acylated ghrelin response to one session circuit resistance exercise in fasted and high carbohydrate meal in healthy young men. Int J Endocrinol Metab.

[166] Hagobian T.A., Sharoff C.G., Stephens B.R., Wade G.N., Silva J.E., Chipkin S.R. (2009). Effects of exercise on energy-regulating hormones and appetite in men and women. Am J Physiol Regul Integr Comp Physiol.

[167] Cairns S.P. (2006). Lactic acid and exercise performance : culprit or friend?. Sports Med.

[168] Overgaard M., Rasmussen P., Bohm A.M., Seifert T., Brassard P., Zaar M. (2012). Hypoxia and exercise provoke both lactate release and lactate oxidation by the human brain. Faseb J.

[169] Park J., Kim J., Mikami T. (2021). Exercise-induced lactate release mediates mitochondrial biogenesis in the hippocampus of mice via monocarboxylate transporters. Front Physiol.

[170] Lhomme T., Clasadonte J., Imbernon M., Fernandois D., Sauve F., Caron E. (2021). Tanycytic networks mediate energy balance by feeding lactate to glucose-insensitive POMC neurons. J Clin Invest.

[171] Ordenes P., Villar P.S., Tarifeno-Saldivia E., Salgado M., Elizondo-Vega R., Araneda R.C. (2021). Lactate activates hypothalamic POMC neurons by intercellular signaling. Sci Rep.

[172] Chen Y., Zhang S., Ye L., Chen H., Yu L., Wu D. (2023). An acute bout of exercise suppresses appetite via central lactate metabolism. Neuroscience.Volume.

[173] Lund J., Breum A.W., Gil C., Falk S., Sass F., Isidor M.S. (2023). The anorectic and thermogenic effects of pharmacological lactate in male mice are confounded by treatment osmolarity and co-administered counterions. Nat Metab.

[174] Feng Q., Liu Z., Yu X., Huang T., Chen J., Wang J. (2022). Lactate increases stemness of CD8 + T cells to augment anti-tumor immunity. Nat Commun.

[175] Racotta R., Russek M. (1977). Food and water intake of rats after intraperitoneal and subcutaneous administration of glucose, glycerol and sodium lactate. Physiol Behav.

[176] Jansen R.S., Addie R., Merkx R., Fish A., Mahakena S., Bleijerveld O.B. (2015). N-lactoyl-amino acids are ubiquitous metabolites that originate from CNDP2-mediated reverse proteolysis of lactate and amino acids. Proc Natl Acad Sci U S A.

[177] Li V.L., He Y., Contrepois K., Liu H., Kim J.T., Wiggenhorn A.L. (2022). An exercise-inducible metabolite that suppresses feeding and obesity. Nature.

[178] Gonzalez R.R., Berglund L.G., Gagge A.P. (1978). Indices of thermoregulatory strain for moderate exercise in the heat. J Appl Physiol Respir Environ Exerc Physiol.

[179] Kamon E., Pandolf K., Cafarelli E. (1974). The relationship between perceptual information and physiological responses to exercise in the heat. J Hum Ergol Tokyo.

[180] Kunstetter A.C., Wanner S.P., Madeira L.G., Wilke C.F., Rodrigues L.O., Lima N.R. (2014). Association between the increase in brain temperature and physical performance at different exercise intensities and protocols in a temperate environment. Braz J Med Biol Res.

[181] Nybo L., Secher N.H., Nielsen B. (2002). Inadequate heat release from the human brain during prolonged exercise with hyperthermia. J Physiol.

[182] Lima P.M.A., Campos H.O., Foscolo D.R.C., Szawka R.E., Wanner S.P., Coimbra C.C. (2019). The time-course of thermoregulatory responses during treadmill running is associated with running duration-dependent hypothalamic neuronal activation in rats. Brain Struct Funct.

[183] Fonseca C.G., Pires W., Lima M.R.M., Guimaraes J.B., Lima N.R.V., Wanner S.P. (2014). Hypothalamic temperature of rats subjected to treadmill running in a cold environment. PLoS One.

[184] Kaiyala K.J., Morton G.J., Thaler J.P., Meek T.H., Tylee T., Ogimoto K. (2012). Acutely decreased thermoregulatory energy expenditure or decreased activity energy expenditure both acutely reduce food intake in mice. PLoS One.

[185] Deem J.D., Faber C.L., Pedersen C., Phan B.A., Larsen S.A., Ogimoto K. (2020). Cold-induced hyperphagia requires AgRP neuron activation in mice. Elife.

[186] Molinas A.T.R., Desmoulins L.D., Hamling B.V., Butcher S.M., Anwar I.J., Miyata K. (2019). Interaction between TRPV1-expressing neurons in the hypothalamus. J Neurophysiol.

[187] Varela L., Horvath T.L. (2019). Parallel paths in PVH control of feeding. Neuron.

[188] Tong Q., Ye C., McCrimmon R.J., Dhillon H., Choi B., Kramer M.D. (2007). Synaptic glutamate release by ventromedial hypothalamic neurons is part of the neurocircuitry that prevents hypoglycemia. Cell Metab.

[189] Vong L., Ye C., Yang Z., Choi B., Chua S., Lowell B.B. (2011). Leptin action on GABAergic neurons prevents obesity and reduces inhibitory tone to POMC neurons. Neuron.

[190] Ansdell P., Thomas K., Hicks K.M., Hunter S.K., Howatson G., Goodall S. (2020). Physiological sex differences affect the integrative response to exercise: acute and chronic implications. Exp Physiol.

[191] Yale J.F., Leiter L.A., Marliss E.B. (1989). Metabolic responses to intense exercise in lean and obese subjects. J Clin Endocrinol Metab.

[192] Riddell M.C., Peters A.L. (2022). Exercise in adults with type 1 diabetes mellitus. Nat Rev Endocrinol.

[193] Sato S., Dyar K.A., Treebak J.T., Jepsen S.L., Ehrlich A.M., Ashcroft S.P. (2022). Atlas of exercise metabolism reveals time-dependent signatures of metabolic homeostasis. Cell Metabol.

[194] Xu B., Kalra P.S., Farmerie W.G., Kalra S.P. (1999). Daily changes in hypothalamic gene expression of neuropeptide Y, galanin, proopiomelanocortin, and adipocyte leptin gene expression and secretion: effects of food restriction. Endocrinology.

[195] Van Drunen R., Eckel-Mahan K. (2021). Circadian rhythms of the hypothalamus: from function to physiology. Clocks Sleep.

[196] Mandelblat-Cerf Y., Ramesh R.N., Burgess C.R., Patella P., Yang Z., Lowell B.B. (2015). Arcuate hypothalamic AgRP and putative POMC neurons show opposite changes in spiking across multiple timescales. Elife.

[197] Sylow L., Kleinert M., Richter E.A., Jensen T.E. (2017). Exercise-stimulated glucose uptake - regulation and implications for glycaemic control. Nat Rev Endocrinol.

